# Florence Nightingale and the War

**Published:** 1916-02-26

**Authors:** Florence Nightingale


*To the Editor of* The Hospital.

### Florence Nightingale and the War.

*To the Editor of* The Hospital.

Mr. J. Vine Milne has sent to the *Morning Post* the following letter from Miss Nightingale, which was written fifteen years ago to the President of the Balaclava Society (that is to say, during the South African War, to which the text evidently refers). His comment is: "Here are the sentiments of an Englishwoman, one of the noblest of her race, the most self-sacrificing, the tenderest—a woman fit to join hands with Joan of Arc."

10 South Street, Park Lane, W., October 24.

Dear Sir,—Though I am not able to be with you in presence at your annual commemoration, yet my heart and soul are with you. How pleased I am, though ill, to be able to write a few lines to you. I thank you with all my heart for your kind thoughts of me. I wish I could say, as we thought a few days ago we might have said, that there would be peace.

But still, as was once written about the advantages of persecution, we may write about the advantages of war, yet few men, and perhaps no women, have seen as much as I have of the horrors of war. But see those manly fellows in time of war, men not near the beasts, as sometimes we too sadly see in the time of peace; see them not one taking a drop too much; not one gallivanting with the women; everyone devoting, aye, even his life for his comrade, fetching his comrade off the field, without notice or praise from anyone, either in words or in print; and if killed in the attempt his name only goes down as "killed in battle"; always devoted even to the death, as our Great Master and Friend, Jesus Christ, was to His fellow men.

Oh, if such be war, we will not say, "Let there always be war!" but blessed be war which makes such heroes of fellowship out of war. Sad is the death of our comrades. But we may say, "Death comes not untimely to him who is fit to die. The briefer life, the earlier immortality." And who would keep him back? Not even his wife. My friends, survivors of Balaclava, I pledge you in this cup, not all of grief, but of living life, worth perhaps all the downy chairs we know of. Those who are gone are with us still, working with us at the good and right and the happiness of our fellow men.—Pray believe me ever your faithful friend,

Florence Nightingale.

